# The Ministry and Its Ministers

**Published:** 1923-10

**Authors:** 


THE
### HOSPITAL and HEALTH
REVIEW

### THE MINISTRY AND ITS MINISTERS.

THERE is a touch of the grotesque about the mere statement of the fact that there have been three Ministers of Health in less than nine months, and four in less than twelve. Sir Arthur Griffith-Boscawen was the luckless victim of an election which forbade his acquisition of a seat in Parliament. He was sacrificed to the wobbling of the Government on the housing question; just as, a short time before, he had been sacrificed at the polls to the wobbling of its predecessor on the regulation of agriculture. His successor, Mr. Neville Chamberlain (who has held three Ministerial posts since the year began), was allowed just enough time to get through a reasonable Housing Act, and now, for his just steerage at the Ministry of Health, he has been set to the even more arduous task of managing the national finances. To him has succeeded Sir W. Joynson-Hicks, whose rise in administrative rank has also been rapid. Happily for him and for the country, the new Minister of Health—the fifth since the creation of the office in 1919—has had the advantage of the Parliamentary recess in which to obtain a preliminary view of the important and far-reaching work that awaits him. As a Minister and an administrator Sir W. Joynson-Hicks is comparatively an untried man, and neither he nor we can blink the fact that the great department over which he now presides will be a severe test of his capacity and adaptability. He goes to Whitehall with the general good wishes, and the sincere hope that he will so conduct it as to bring it substantially nearer to the ideal with which it was created.

But not even a Heaven-sent Minister could raise the central Health Department of this country to the position it ought to occupy if he were allowed to hold office for a mere matter of three, or perhaps six, months. In face of these rapid changes not only is continuity of policy impossible, but vigour and initiative are out of the question. The fleeting incumbent of the office is fortunate if he is able to acquire a preliminary general acquaintance with the multitudinous activities of a department the working of which affects, more or less closely, every individual in the country. It is clear that we cannot continue to have for our Ministers of Health a series of transient and embarrassed phantoms at the mercy of every rearrangement of portfolios. It is bad enough that the Ministry of Agriculture should be looked upon as a stepping-stone to something more dignified and better paid; it would be intolerable that the great work of improving the health of the country by encouraging preventive medicine, getting rid of slums, providing sufficient and decent housing, subsidising research into the cause and treatment of disease, and the hundred other activities of a department as far-reaching as it is vital, should be jeopardised for the convenience of Governments which may be here to-day and gone to-morrow. At present these things are actually being jeopardised. No Minister of Health can sit down quietly and, in consultation with the permanent heads of his departments, elaborate a rational and continuing programme. Indeed, were it not that these departmental chiefs are men of sagacity and experience, the

Ministry would by now have been a mere slice of chaos.

There is obviously something wrong in a political system that inflicts such disabilities upon an organisation which lies at the root of the physical well-being of the nation. Only the most exceptional and forceful men can continue to be efficient when they are moved about from office to office every few months, and most Ministers are not exceptional. They are usually men of average ability who owe their position to party exigencies, perhaps even to mere Parliamentary convenience. It is absurd, on the face of it, that a Minister of Health should be a political personage at all, but we might as well cry for the moon as expect any Prime Minister to denude himself of so important a piece of preferment. It does not follow that because a Cabinet Minister may be a wise counsellor in an international dispute he is fitted to deal with great medical, scientific, and social problems—these Admirable Crichtons are much more rare than black swans. At present the public at large have hardly begun to realise how momentous are the issues involved in this matter. When they do realise them they may be expected to protest vigorously against this playing fast and loose with interests which are the intimate concern of each one of us.

